# Circulating Citrate Is Reversibly Elevated in Patients with End-Stage Liver Disease: Association with All-Cause Mortality

**DOI:** 10.3390/ijms252312806

**Published:** 2024-11-28

**Authors:** Yakun Li, Mateo Chvatal-Medina, Maria Camila Trillos-Almanza, Arno R. Bourgonje, Margery A. Connelly, Han Moshage, Stephan J. L. Bakker, Vincent E. de Meijer, Hans Blokzijl, Robin P. F. Dullaart

**Affiliations:** 1 Department of Gastroenterology and Hepatology, University Medical Center Groningen, University of Groningen, 9700 RB Groningen, The Netherlands; y.li01@umcg.nl (Y.L.); m.chvatal-medina@umcg.nl (M.C.-M.); m.c.trillos.almanza@umcg.nl (M.C.T.-A.); a.r.bourgonje@umcg.nl (A.R.B.); a.j.moshage@umcg.nl (H.M.); h.blokzijl@umcg.nl (H.B.); 2 The Henry D. Janowitz Division of Gastroenterology, Department of Medicine, Icahn School of Medicine at Mount Sinai, New York, NY 10029, USA; 3 Labcorp, 100 Perimeter Park, Morrisville, NC 27560, USA; connem5@labcorp.com; 4 Department of Internal Medicine, Division of Nephrology, University Medical Center Groningen, University of Groningen, 9700 RB Groningen, The Netherlands; s.j.l.bakker@umcg.nl; 5 Department of Surgery, Division of Hepato-Pancreato-Biliary Surgery and Liver Transplantation, University Medical Center Groningen, University of Groningen, 9700 RB Groningen, The Netherlands; v.e.de.meijer@umcg.nl; 6 Department of Internal Medicine, Division of Endocrinology, University Medical Center Groningen, University of Groningen, 9700 RB Groningen, The Netherlands

**Keywords:** plasma citrate, end-stage liver disease, liver transplantation, all-cause mortality

## Abstract

Circulating citrate may serve as a proxy for mitochondrial dysfunction which plays a role in the progression of end-stage liver disease (ESLD). This study aimed to determine the extent of alterations in circulating citrate in patients with ESLD, and examined its association with all-cause mortality among ESLD patients while on the waiting list for liver transplantation. Plasma citrate levels were measured using nuclear magnetic resonance spectroscopy in 129 ESLD patients (TransplantLines cohort study; NCT03272841) and compared to levels in 4837 participants of the community-dwelling PREVEND cohort. Plasma citrate levels were 40% higher in ESLD patients compared to PREVEND participants (*p* < 0.001). In a subset of 30 ESLD patients, citrate decreased following liver transplantation (*p* < 0.001), resulting in levels that were slightly lower than those observed in PREVEND participants. In multivariable analysis, plasma citrate levels were positively associated with Child–Turcotte–Pugh classification and inversely associated with estimated glomerular filtration rate (both *p* < 0.05). Survival was significantly reduced in ESLD patients in the highest citrate tertile (log-rank *p* = 0.037). Elevated citrate levels were associated with an increased risk of all-cause mortality in ESLD patients (HR per 1 Ln SD increment: 1.65 [95% CI: 1.03–2.63], *p* = 0.037). This association was suggested to be particularly present in men (HR: 2.04 [95% CI: 1.08–3.85], *p* = 0.027). In conclusion, plasma citrate levels are elevated in ESLD patients and decrease following liver transplantation. Moreover, elevated plasma citrate levels may be associated with increased all-cause mortality in ESLD patients, likely more pronounced in men.

## 1. Introduction

End-stage liver disease (ESLD) represents an important global health burden, characterized by high mortality rates and a severe impact on patient quality of life [1,2]. Liver transplantation (LT) remains the only lifesaving treatment available, providing substantial improvement in survival and quality of life [3]. Despite notable advancements in medical therapies and management strategies, mortality among ESLD patients has continued to be elevated over the past few decades [4]. ESLD encompasses a broad spectrum of liver pathologies that collectively lead to liver failure, with mitochondrial dysfunction playing a critical role in its pathogenesis [5,6,7].

Impaired mitochondrial function leads to reduced energy production and an imbalance in redox state, which adversely affects cell survival by changing metabolism and subcellular transport [5]. These changes weaken the resilience of hepatocytes by impairing ATP generation and altering metabolism, making them more vulnerable to external detrimental factors such as toxins, infections, and inflammatory processes. Over time, these disruptions hinder the liver’s ability to repair and regenerate, thereby accelerating disease progression and contributing to liver failure [7]. As mitochondrial dysfunction is essential to the deterioration seen in ESLD, there is growing interest in identifying biomarkers that reflect mitochondrial activity and could indicate disease severity and prognosis.

Among other emerging biomarkers, circulating citrate has gained attention as a promising indicator of disease progression [8] and mortality risk [9]. Citrate is a critical metabolite in the tricarboxylic acid (TCA) cycle, an essential pathway for energy production through the oxidation of acetyl-CoA derived from fats, proteins, and carbohydrates. This cycle also serves as a hub for amphibolic metabolism, integrating anabolic and catabolic processes [10,11]. Using nuclear magnetic resonance (NMR) spectroscopy, recent studies have identified plasma citrate as one of the potential biomarkers predictive of all-cause mortality [12,13]. In addition, it has been suggested that elevated plasma citrate levels may be linked to an increased risk of cardiovascular mortality in patients with type 2 diabetes [9]. In the context of liver disease, circulating citrate levels have been associated with liver fibrosis in conditions such as non-alcoholic fatty liver disease (NAFLD; new nomenclature: metabolic dysfunction-associated steatotic liver disease or MASLD) and non-alcoholic steatohepatitis (NASH- new nomenclature: metabolic dysfunction-associated steatohepatitis or MASH) [8]. However, the relationship between circulating citrate concentrations and mortality in patients with ESLD remains unknown.

Therefore, we initiated this study on plasma concentrations of citrate in patients with ESLD on the transplant waiting list. We aimed to investigate (1) to what extent plasma citrate could be elevated in end-stage liver disease and to reveal the reversibility of citrate alterations after transplantation, (2) the determinants of plasma citrate in ESLD patients, and (3) the longitudinal association of citrate with all-cause mortality in these patients.

## 2. Results

### 2.1. Comparison of Baseline Clinical and Laboratory Characteristics Between Patients with End-Stage Liver Disease and PREVEND Participants

A total of 129 patients with ESLD participated in the study. Their clinical and laboratory characteristics were compared with 4837 participants from the Prevention of Renal and Vascular End-stage Disease (PREVEND) study (Table 1). Average age was higher in the ESLD patients compared to the PREVEND group (*p* < 0.001). Male predominance was noted in the ESLD group compared to the PREVEND cohort (*p* < 0.001). ESLD patients had a higher body mass index (BMI) (*p* < 0.001). Lifestyle factors varied, with only 12.4% of ESLD patients currently smoking compared to 27.3% in PREVEND (*p* < 0.001); alcohol consumption was also lower in ESLD patients. Diabetes prevalence was substantially higher in ESLD versus PREVEND participants (*p* < 0.001). Use of glucose-lowering, lipid-lowering, and antihypertensive drugs was higher in ESLD patients (*p* < 0.05). Total cholesterol, serum creatinine, and hemoglobin levels were lower in ESLD patients, whereas fasting glucose, total bilirubin, alanine aminotransferase (ALT), aspartate aminotransferase (AST), gamma-glutamyl transferase (GGT), and alkaline phosphatase (ALP) levels were higher in ESLD patients (*p* < 0.001).

As shown in Table 1, plasma citrate levels in ESLD patients were significantly higher compared to PREVEND participants (median 153 [118–195] vs. 106.2 [91.1–122.8] µmol/L, *p* < 0.001), corresponding to a 40% higher concentration among ESLD patients. After propensity score matching for age, sex, BMI, history of cardiovascular disease and diabetes, citrate levels were still elevated in ESLD patients compared to their matched PREVEND control subjects (*n* = 129) (median 153 [118–195] vs. 106.2 [93.2–124.9] µmol/L, *p* < 0.001).

**Figure 1:**
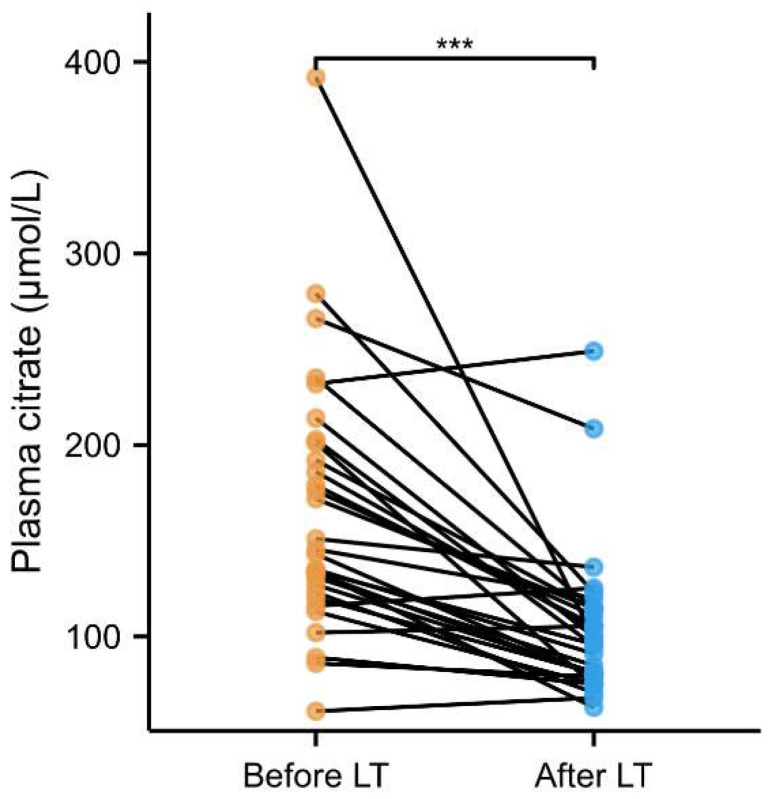
Paired comparison of plasma citrate among 30 patients with end-stage liver disease, before and after liver transplantation. *** *p* < 0.001. LT: liver transplantation.

**Figure 2:**
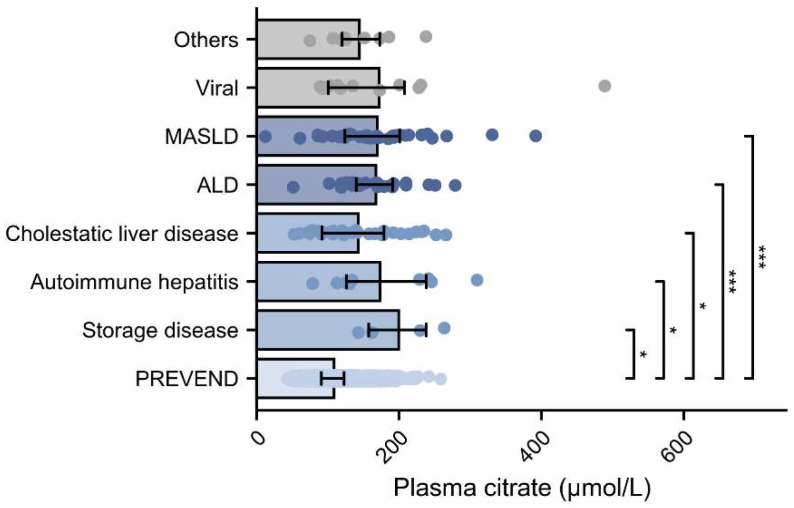
Plasma citrate concentrations according to etiology vs. PREVEND. *** *p* < 0.001, * *p* < 0.05. Gray represents categories with no statistical significance, medium blue represents * (*p* < 0.05), and dark blue represents *** (*p* < 0.001). ALD: alcohol-associated liver disease; MASLD: metabolic dysfunction-associated steatotic liver disease; PREVEND: prevention of renal and vascular end-stage disease.

Of the ESLD patients, 30 were studied again at least 1 year after LT. In these patients, median plasma citrate decreased from 144.2 [124–198.8] to 97.5 [80–114.4] µmol/L (*p* < 0.001), levels that were significantly lower than PREVEND participants (*p* = 0.032) (Figure 1). When compared with PREVEND participants, citrate concentrations were elevated in patients with storage disease, autoimmune hepatitis, cholestatic liver disease, alcohol-associated liver disease (ALD), and MASLD as underlying etiology, as illustrated in Figure 2.

Table 2 shows baseline characteristics of ESLD patients categorized by tertiles of plasma citrate levels. Those in the highest citrate tertile were older, used antihypertensives more frequently, and had higher serum creatinine, as well as lower total cholesterol and estimated glomerular filtration rate (eGFR) compared to those in lower tertiles (Table 2).

### 2.2. Associations of Plasma Citrate with Clinical and Laboratory Variables in Patients with End-Stage Liver Disease

**Figure 3:**
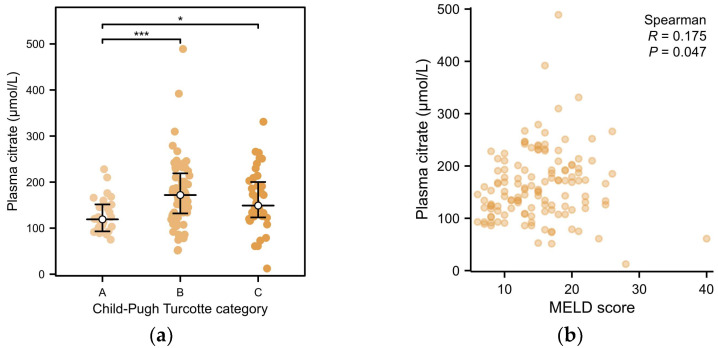
Plasma concentrations of citrate according to Child–Turcotte–Pugh classification and MELD scores in 129 patients with ESLD. (**a**) Plasma citrate concentrations according to Child–Turcotte–Pugh classification. (**b**) Relationship of plasma citrate concentrations with MELD score (Spearman rank correlation coefficient: 0.175, *p* = 0.047). Data are presented as medians with interquartile ranges. *** *p* < 0.001, * *p* < 0.05. MELD: model for end-stage liver disease; ESLD: end-stage liver disease.

In univariable analysis, plasma citrate levels were associated with the Child–Turcotte–Pugh (CTP) classification (Figure 3a). Additionally, a weak but significant association between plasma citrate and the model for end-stage liver disease (MELD) score was observed (Figure 3b). Multivariable linear regression analyses were performed to identify relevant clinical and laboratory variables that may be associated with citrate levels. As demonstrated in Table 3, multivariable linear regression analyses in ESLD patients showed an inverse association of citrate with eGFR, which remained significant after adjustment for age, sex, use of antihypertensives, CTP classification, and MELD score. Moreover, plasma citrate remained positively associated with CTP classification in the fully adjusted model (Table 3).

### 2.3. Longitudinal Analysis of Plasma Citrate with All-Cause Mortality in End-Stage Liver Disease Patients on the Waiting List

**Figure 4:**
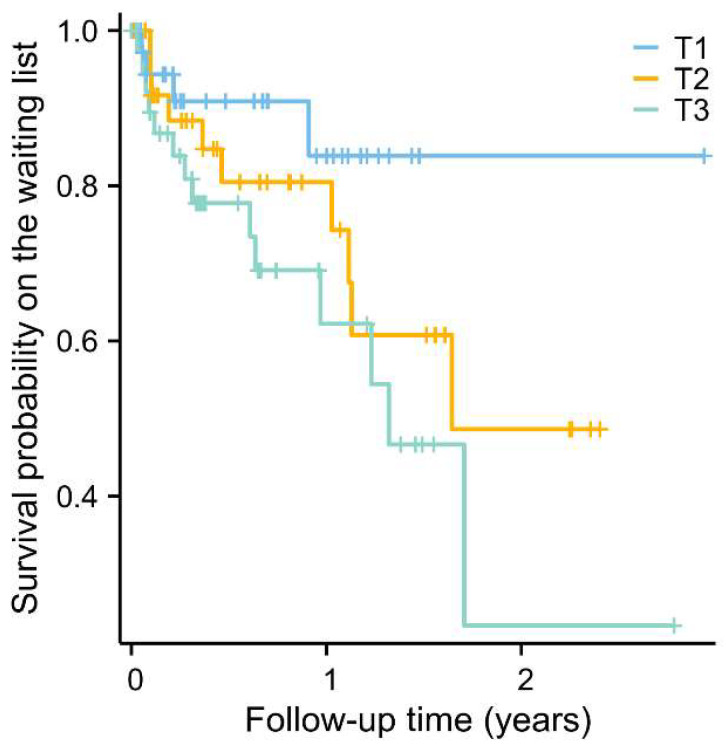
Kaplan–Meier survival curves for the association between plasma citrate levels and the risk of all-cause mortality in end-stage liver disease patients on the waiting list (T1: <126 μmol/L; T2: 126–179 μmol/L; T3: >179 μmol/L) (log-rank test, T1 vs. T3 HR: 3.05 [1.21–7.69], *p* = 0.037).

Of the 129 ESLD patients, 29 (22.5%) died during a follow-up of 140 (IQR 52–381) days. Figure 4 presents the Kaplan–Meier curves for all-cause mortality according to tertiles of citrate. Survival was lowest among ESLD patients with the highest citrate levels (T3 vs. T1: log-rank test, *p* = 0.037).

In Cox proportional hazards regression analyses, plasma citrate levels were associated with increased all-cause mortality risk in ESLD patients (Table 4, Model 1, HR per 1 Ln SD increment: 1.65 [95% CI: 1.03–2.63], *p* = 0.037). The association was also observed in the highest citrate tertile compared to the lowest tertile (HR: 3.13 [95%CI: 1.03–9.54], *p* = 0.044). However, this association did not reach formal statistical significance after adjusting for age and sex (Table 4, Model 2), or eGFR and CTP classification (Table 4, Model 3 and 4). In sex-stratified Cox proportional hazards regression analyses, an association was observed between plasma citrate levels and increased mortality risk among males in unadjusted and age-adjusted analyses. However, this association was not evident in females.

## 3. Discussion

The present study demonstrates that plasma citrate concentrations are on average 40% higher in patients with ESLD compared to the levels found in the general population. In a small subset of patients who were followed after LT, plasma citrate decreased to levels that were slightly lower than those in the general population, supporting the possibility that such citrate elevations are reversible.

Higher circulating citrate concentrations were found to be associated with an increased risk of all-cause mortality in crude analysis. This association varied between sexes and was suggested to be present in men only. Our results raise the possibility of a potential pathogenic involvement of citrate-related pathways, reflected by plasma citrate levels, in the mortality risk of ESLD patients on the waiting list for liver transplantation.

We observed that citrate levels were elevated in patients with ESLD but decreased following liver transplantation. Maintaining normal plasma citrate levels is crucial for both humans and animals, as it is necessary for many physiological processes [14]. Under normal conditions, citrate is retained in the mitochondria, where it enters the Krebs cycle and is used for ATP production and the generation of substrates for various metabolic pathways [11]. In ESLD patients, this elevation is likely due to not only enhanced release from damaged hepatocytes but also from reduced uptake by the liver. Renal clearance plays a major role in the removal of citrate from the body [14]. Impaired kidney function is common in ESLD and can further reduce citrate excretion. In our study, we observed that patients with the highest citrate tertile had lower eGFR levels. Accordingly, plasma citrate was inversely associated with eGFR in multivariable analysis. The inverse association between citrate and kidney function suggests that renal excretion may be a relevant factor in regulating plasma citrate levels in ESLD patients. Additionally, some studies have suggested that the gut microbiota may serve as a source of circulating TCA cycle intermediates [15]. For example, elevated circulating succinate levels in human obesity have been linked to specific gut microbiota [16]. Given that patients with cirrhosis often have imbalances in their microbiome [17,18], we hypothesize that elevated citrate levels may also be linked, to some extent, to gut microbiota dysregulation. Following liver transplantation, the newly transplanted liver restores normal metabolic and clearance functions, reducing the abnormal accumulation of citrate.

Citrate has been shown to be associated with incident mortality in various populations [12,13]. In a large-scale study of the general population, citrate was identified as one of four biomarkers, selected from 106 circulating NMR-measured metabolites, that predicted cardiovascular mortality, cancer-related death, and all-cause mortality [12]. Additionally, elevated citrate levels measured at admission have been linked to an increased 3-month mortality rate in patients with acute heart failure [19]. Furthermore, plasma citrate levels have been associated with a higher risk of cardiovascular mortality in patients with type 2 diabetes [9]. However, a prospective study investigating the relationship between TCA cycle components and mortality risk following an acute coronary syndrome found no association between citrate and cardiovascular outcomes. Instead, positive associations were observed with other metabolites such as isocitrate, aconitate, and d/l-2-hydroxyglutarate [20]. We surmise that citrate may have predictive value only in specific subgroups of populations, rather than serving as a universal biomarker in all categories.

A notable finding in our study is that the association between citrate and all-cause mortality in ESLD patients was observed in males but not significantly so in females. Such a sex-specific mortality pattern has also been noted in previous research. For example, increased plasma citrate levels have been linked to more advanced stages of liver cirrhosis, particularly in males [8]. Additionally, another study found that the association between citrate and cardiovascular mortality in patients with type 2 diabetes was also male-specific [9]. A study in rats demonstrated early diet-related changes in mitochondrial function in males, but not in females [21]. Similarly, a large-scale study in mice identified sex differences in mitochondrial function, with male mice showing reduced performance compared to females, potentially due to genetic factors [22]. In humans, research has revealed novel sex-specific associations involving lipid species that play a role in mitochondrial fatty acid transport, β-oxidation, and TCA cycle flux [23]. Nonetheless, the underlying mechanisms remain poorly understood. Future studies with larger cohorts are needed to confirm these sex-specific findings and further explore the underlying biological mechanisms.

Strengths of our study include its role as the first investigation to systematically evaluate the clinical impact of plasma citrate in this patient population, including evaluation regarding mortality on the waiting list, with a detailed and standardized assessment of clinical and laboratory characteristics consequent to the TransplantLines Biobank and Cohort study set-up. We compared plasma citrate with participants from the PREVEND study, which served as a large community-dwelling control cohort from the same region of The Netherlands. Our study’s limitations also warrant recognition. The cohorts largely consisted of a Western European population, which may limit the generalizability of our findings to other ethnic groups. The observational nature of our study precludes inference to causality. Furthermore, we lacked longitudinal assessments of plasma citrate, which could have provided more insight into changes over time. We also lacked data on some potentially relevant confounders, such as the habitual dietary intake of patients. Finally, the mortality analysis should be considered preliminary due to the comparatively low median MELD score and the limited number of ESLD patients, especially the rather small number of females. More comprehensive, multicenter cohort studies are necessary to validate the findings, alongside longitudinal studies to assess variations in plasma citrate levels over time and their relationship to clinical outcomes. Integrating plasma citrate measurements into existing prognostic models may further clarify its predictive value.

In conclusion, plasma citrate levels, as a proxy for disturbances in TCA or citric acid cycle and mitochondrial dysfunction, are elevated in ESLD patients and likely return to normal levels after liver transplantation. We suggest that elevated circulating citrate levels may be associated with an increased risk of all-cause mortality in patients with ESLD on the transplant waiting list, particularly in males. Future research is needed to elucidate the underlying mechanisms driving this association.

## 4. Materials and Methods

### 4.1. Study Population

This study was conducted following the STROBE (Strengthening the Reporting of Observational Studies in Epidemiology) guidelines [24]. Patients with ESLD were selected from the TransplantLines cohort, a prospective observational study based at the University Medical Center Groningen (UMCG), The Netherlands (NCT03272841) [25]. The study was conducted in compliance with the Declaration of Helsinki [26]. Exclusion criteria included inability to understand the Dutch language, cognitive impairment that hinders understanding of questionnaires or physical tests, prior re-transplantation, and lack of citrate measurements.

For the control cohort, data were extracted from the PREVEND study [27,28], a population-dwelling cohort in Groningen, The Netherlands. The PREVEND study, initiated between 1997 and 1998, has been described in detail elsewhere [27,28]. In brief, Groningen residents aged between 28 and 75 years of age were invited to submit a morning urine sample and complete a demographic and cardiovascular history questionnaire. Pregnant women and those with insulin-dependent diabetes were excluded. Participants with a urinary albumin concentration of ≥10 mg/L, along with a randomly selected control group with lower concentrations, were invited for further evaluation. For the current analysis, we focused on those who completed the second screening round between 2001 and 2003. Participants enrolled in this analysis were verified to be free of liver disease (based on a questionnaire and medical records obtained from primary care physicians), and had available plasma citrate measurements. The PREVEND study was approved by the UMCG Medical Ethics Committee (MEC96/01/022) and conducted according to the Declaration of Helsinki [26]. Written informed consent was secured from all participants in both the TransplantLines and PREVEND studies. A Consort flow chart of the study participants from the TransplantLines and the PREVEND cohorts is shown in Supplementary Figure S1.

### 4.2. Data Collection and Clinical Measurements

The TransplantLines study, initiated in June 2015, continuously collects data on transplant candidates with ESLD. For the current study, data were collected up to June 2021. During outpatient visits, questionnaires and blood samples were obtained from all patients with standardized procedures [25]. Participants maintained their regular medication on the day of blood collection. Anthropometric measurements were recorded with a standardized protocol. Patient information, including weight, height, BMI, blood pressure, smoking status, alcohol consumption (standardized to 10 g per drink in The Netherlands), medication use (glucose and lipid-lowering drugs, antihypertensive medication), and medical histories such as cardiovascular disease (CVD) and diabetes (defined as fasting plasma glucose >7.0 mmol/L, non-fasting plasma glucose >11.1 mmol/L, a self-reported diagnosis, or the use of glucose-lowering drugs), was extracted from the TransplantLines Biobank. Blood pressure readings were taken multiple times to ensure reliability. The eGFR was calculated using the 2012 CKD Epidemiology Collaboration creatinine-based formula [29]. Additional review of electronic patient records of study participants was performed to obtain data concerning the etiology of liver disease, including storage diseases (e.g., Wilson’s disease, hemochromatosis and alpha-1 antitrypsin deficiency), autoimmune hepatitis, cholestatic liver diseases (e.g., primary sclerosing cholangitis, primary biliary cholangitis), viral infections (e.g., hepatitis B virus, hepatitis C virus), ALD, MASLD, and others (e.g., vascular diseases). Assessments based on imaging, histology, or transient elastography, along with biochemical and clinical variables, were used to compute the MELD scores and CTP classification to evaluate the severity of ESLD. The MELD score was calculated by serum total bilirubin, creatinine, and the international normalized ratio (INR) [30]. The CTP classification was calculated by total bilirubin, serum albumin, INR, presence of ascites, and hepatic encephalopathy [31]. Data on mortality were obtained from electronic patient records and verified by the Dutch Central Bureau of Statistics.

In the PREVEND cohort, data were collected on demographics, lifestyle factors, anthropometric measurements, medical history, and medication use, which was combined with information from a pharmacy-dispensing registry as previously described [28].

### 4.3. Laboratory Measurements

Venous blood samples were collected from participants in both TransplantLines and PREVEND cohorts after an overnight fast. Laboratory methods for PREVEND are reported as described in detail previously [27]. A panel of standardized laboratory assays, including serum ALT, AST, GGT, ALP, total bilirubin, albumin (only available in the TransplantLines cohort), serum creatinine, hemoglobin, thrombocytes, leucocytes (only available in the TransplantLines cohort), glycated hemoglobin (HbA1c; only available in the TransplantLines cohort), and plasma glucose, were analyzed with standardized laboratory measurements and quality assessment control at the department of Laboratory Medicine of the University Medical Center Groningen, The Netherlands.

Ethylenediaminetetraacetic acid (EDTA)-anticoagulated plasma samples were centrifuged at 1400 g for 15 min at 4 °C and then stored at −80 °C. Plasma samples were shipped to Labcorp (Morrisville, NC, USA) and analyzed on the Vantera^®^ Clinical Analyzer. Plasma samples were prepared on board the instrument and automatically delivered to the flow probe in the NMR spectrometer’s magnetic field. Total cholesterol was measured as previously described [32]. Citrate levels were determined using NMR spectroscopy as previously described [9]. The stability of citrate has been established in samples that were frozen for up to 12 years at temperatures below −70 °C. Inter-assay precision for citrate, expressed as coefficients of variation (%CV), ranged from 5.2% for high concentration samples to 9.6% for low concentration samples.

### 4.4. Statistical Analysis

Statistical analyses were carried out using IBM SPSS software (version 25.0, IBM Corp, Armonk, NY, USA) and R software (version 4.2.1, Vienna, Austria). Significance was set at a two-sided *p*-value of less than 0.05. Continuous variables were presented as mean ± standard deviation (SD) for normally distributed data, or as medians with interquartile range (IQR) for non-normally distributions, while categorical variables were reported as frequencies and percentages.

The citrate concentrations were categorized into three tertiles (T1: <126 μmol/L; T2: 126–179 μmol/L; T3: >179 μmol/L). Baseline characteristics were compared between ESLD patients and PREVEND participants, or across tertiles. For normally distributed continuous variables, differences between two groups were evaluated using independent *t*-tests, and across three groups with one-way analysis of variance (ANOVA). Non-normally distributed variables were assessed using the Mann–Whitney U-test for two groups, and the Kruskal–Wallis test for three groups. Categorical variables were analyzed with the chi-square test or Fisher’s exact test, depending on expected frequencies. Changes in citrate levels within individuals over time were evaluated using the Wilcoxon signed-rank test. To further compare citrate levels between ESLD patients and PREVEND participants, propensity score matching was used to control for age, sex, BMI, history of cardiovascular disease, and diabetes, with a matching tolerance of ≤0.2 to improve precision.

The relationship between citrate levels and MELD scores in the ESLD group was assessed using Spearman correlation coefficients. Multivariable linear regression analysis was employed to investigate the associations between clinical or laboratory variables and citrate levels. The identified variables were subsequently adjusted for in the Cox regression model to control for potential confounding effects.

To assess the survival distributions across tertiles of citrate levels, Kaplan–Meier curves were generated, and comparisons were made using the log-rank test. Survival time was defined from baseline until the date of the last examination that participants attended, the date of their death, or June 2021 (the final month of follow-up). Univariable and multivariable Cox proportional hazards regression analyses were performed to examine the impact of citrate levels on all-cause mortality, adjusting for potential confounders. Results were reported as hazard ratios (HRs) with corresponding 95% confidence intervals (CIs), with plasma citrate levels being ln-transformed, and HRs expressed in per 1 Ln SD increment. The proportional hazards assumption was tested by examining Schoenfeld residuals to ensure that it was not violated. Additionally, given that previous studies have indicated differences in citrate levels between sexes [8,9], we carried out analyses stratified by sex to explore potential differences in the impact of citrate levels on mortality between sexes.

## Figures and Tables

**Table 1:** Clinical and laboratory characteristics including plasma citrate in patients with end-stage liver disease and PREVEND participants.

	ESLD Patients (*n* = 129)	Before Propensity Score Matching		After Propensity Score Matching	
		PREVEND (*n* = 4837)	*p*-Value	PREVEND (*n* = 129)	*p*-Value
Age (years)	58 ± 10	54 ± 12	<0.001	55 ± 13	0.10
Sex			<0.001		0.06
Male, *n* (%)	84 (65.1)	2388 (49.4)		69 (53.5)	
Female, *n* (%)	45 (34.9)	2449 (50.6)		60 (46.5)	
BMI (kg/m^2^)	28.3 ± 4.8	26.7 ± 4.4	<0.001	27.5 ± 4.9	0.22
SBP (mmHg)	120 ± 18	126 ± 19	0.002	128 ± 21	0.002
DBP (mmHg)	67 ± 11	73 ± 9	<0.001	74 ± 10	<0.001
Current smoking, *n* (%)	16 (12.4)	1321 (27.3)	<0.001	27 (20.9)	0.07
Alcohol consumption (g/day)			<0.001		<0.001
0/rarely, *n* (%)	124 (96.1)	1697 (35.1)		46 (35.7)	
0.1–10, *n* (%)	5 (3.9)	1218 (25.2)		33 (25.6)	
10–30, *n* (%)	0 (0)	993 (20.5)		26 (20.2)	
≥30, *n* (%)	0 (0)	929 (19.2)		24 (18.6)	
Diabetes, *n* (%)	36 (27.9)	294 (6.1)	<0.001	33 (25.6)	0.67
History of cardiovascular disease, *n* (%)	6 (4.7)	301 (6.2)	0.46	15 (11.6)	0.04
Blood glucose-lowering drugs, *n* (%)	35 (27.1)	178 (3.7)	<0.001	18 (14)	0.009
Lipid-lowering drugs, *n* (%)	19 (14.7)	458 (9.5)	0.045	13 (10.1)	0.26
Antihypertensives, *n* (%)	80 (62)	854 (17.7)	<0.001	13 (10.1)	<0.001
Etiology, *n* (%)					
Storage diseases	4 (3.1)				
Autoimmune hepatitis	10 (7.8)				
Cholestatic liver diseases	33 (25.6)				
Viral	12 (9.3)				
ALD	28 (21.7)				
MASLD	33 (25.6)				
Others	9 (7)				
Child–Turcotte–Pugh classification					
A, *n* (%)	28 (21.7)				
B, *n* (%)	63 (48.8)				
C, *n* (%)	38 (29.5)				
MELD score	15 (10, 19)				
Total cholesterol (mmol/L)	3.2 (2.5, 4.1)	5.3 (4.7, 6.1)	<0.001	5.3 (4.7, 6.0)	<0.001
Fasting glucose (mmol/L)	6.2 (5.0, 8.2)	4.8 (4.4, 5.3)	<0.001	5.0 (4.5, 6.1)	<0.001
Serum creatinine (µmol/L)	73.2 (55.6, 95.7)	83.2 (73.9, 92.4)	<0.001	86.2 (76.0, 95.5)	<0.001
eGFR (mL/min/1.73 m^2^)	99.5 (75.5, 109.5)	93.7 (81.6, 104.3)	0.16	91.7 (78.8, 101.4)	0.09
Total bilirubin (µmol/L)	42.0 (23.2, 98.5)	7.0 (5.0, 9.0)	<0.001	6.0 (5.0, 9.0)	<0.001
ALT (U/L)	40.0 (28.0, 60.0)	17.0 (13.0, 24.0)	<0.001	18.0 (13.0, 27.0)	<0.001
AST (U/L)	54.0 (44.0, 84.0)	22.0 (19.0, 26.0)	<0.001	23.0 (20.0, 27.0)	<0.001
GGT (U/L)	95.0 (48.5, 150.5)	24.0 (16.0, 38.0)	<0.001	28.0 (19.0, 41.0)	<0.001
ALP (U/L)	141.0 (98.5, 213.5)	66.0 (55.0, 79.0)	<0.001	70.0 (56.0, 84.0)	<0.001
Hemoglobin (mmol/L)	6.8 (5.8, 7.8)	8.5 (8.0, 9.0)	<0.001	8.6 (7.9, 8.9)	<0.001
Plasma citrate (µmol/L)	153.0 (118.0, 195.0)	106.2 (91.1, 122.8)	<0.001	106.2 (93.2, 124.9)	<0.001

Data are presented as mean ± SD, median (IQR) or as proportions (n) with corresponding percentages (%). ESLD: end-stage liver disease; PREVEND: prevention of renal and vascular end-stage disease; BMI: body mass index; SBP: systolic blood pressure; DBP: diastolic blood pressure; ALD: alcohol-associated liver disease; MASLD: metabolic dysfunction-associated steatotic liver disease; MELD: model for end-stage liver disease; eGFR: estimated glomerular filtration rate; ALT: alanine aminotransferase; AST: aspartate aminotransferase; GGT: gamma-glutamyl transferase; ALP: alkaline phosphatase.

**Table 2:** Baseline characteristics of patients with end-stage liver disease, according to tertiles of plasma citrate levels.

	T1 <126 μmol/L (*n* = 43)	T2 126–179 μmol/L (*n* = 44)	T3 >179 μmol/L (*n* = 42)	*p*-Value
Age (years)	55 (48, 63)	61 (57, 67)	60 (55, 64)	**0.012**
Sex				0.41
Male, *n* (%)	27 (62.8)	32 (72.7)	25 (59.5)	
Female, *n* (%)	16 (37.2)	12 (27.3)	17 (40.5)	
BMI (kg/m^2^)	26.4 (23.3, 29.6)	27.8 (25.5, 31.5)	28.8 (25.0, 32.1)	0.076
SBP (mmHg)	120 (108, 135)	121 (108, 129)	112 (106, 125)	0.34
DBP (mmHg)	69 (61, 75)	64.0 (59, 77)	65 (59, 70)	0.85
Current smoking, *n* (%)	4 (9.3)	5 (11.4)	7 (16.7)	0.57
Alcohol consumption (g/day)				0.063
0/rarely, *n* (%)	39 (90.7)	44 (100)	41 (97.6)	
0.1–10, *n* (%)	4 (9.3)	0 (0)	1 (2.4)	
Diabetes, *n* (%)	9 (20.9)	13 (29.5)	14 (33.3)	0.42
History of cardiovascular disease, *n* (%)	1 (2.3)	4 (9.1)	1 (2.4)	0.35
Blood glucose-lowering drugs, *n* (%)	9 (20.9)	12 (27.3)	14 (33.3)	0.44
Lipid-lowering drugs, *n* (%)	5 (11.6)	9 (20.5)	5 (11.9)	0.42
Antihypertensives, *n* (%)	19 (44.2)	30 (68.2)	31 (73.8)	**0.011**
Etiology, *n* (%)				
Storage diseases	0 (0)	2 (4.5)	2 (4.8)	0.48
Autoimmune hepatitis	3 (7)	3 (6.8)	4 (9.5)	0.83
Cholestatic liver diseases	16 (37.2)	9 (20.5)	8 (19)	0.10
Viral	6 (14)	2 (4.5)	4 (9.5)	0.31
ALD	4 (9.3)	15 (34.1)	9 (21.4)	**0.02**
MASLD	9 (20.9)	11 (25)	13 (31)	0.57
Others	5 (11.6)	2 (4.5)	2 (4.8)	0.47
Child–Turcotte–Pugh classification				**0.003**
A, *n* (%)	17 (39.5)	9 (20.5)	2 (4.8)	
B, *n* (%)	14 (32.6)	23 (52.3)	26 (61.9)	
C, *n* (%)	12 (27.9)	12 (27.3)	14 (33.3)	
MELD score	14 (9, 18)	14 (11, 18)	16 (13, 19)	0.06
Total cholesterol (mmol/L)	3.5 (2.8, 4.7)	2.9 (2.4, 3.7)	3.1 (2.6, 3.7)	**0.032**
Albumin (g/L)	35.0 (29.8, 41.2)	30.0 (27.0, 35.0)	29.5 (27.0, 33.2)	0.05
Fasting glucose (mmol/L)	7.3 (4.6, 8.9)	6.2 (5.0, 7.1)	6.3 (5.3, 8.0)	0.59
HbA1c (%)	5.4 (4.8, 6.3)	5.0 (4.3, 5.6)	4.7 (4.5, 5.5)	0.32
Serum creatinine (µmol/L)	69.6 (50.5, 82.6)	70.2 (55.8, 85.7)	85.8 (64.6, 104.6)	**0.041**
eGFR (mL/min/1.73 m^2^)	102.8 (88.5, 119.3)	102.2 (81.2, 109.4)	84.0 (68.1, 100.6)	**0.003**
Total bilirubin (µmol/L)	49.0 (10.8, 168.8)	32.5 (23.8, 75.0)	53.5 (28.0, 81.8)	0.35
ALT (U/L)	47.0 (32.0, 74.0)	37.5 (28.5, 58.8)	38.0 (27.5, 45.8)	0.25
AST (U/L)	58.0 (37.0, 122.0)	51.0 (43.2, 65.8)	54.0 (44.8, 84.2)	0.55
GGT (U/L)	101.0 (60.0, 255.0)	101.5 (57.5, 149.8)	71.0 (35.5, 135.5)	0.33
ALP (U/L)	129.0 (80.0, 220.0)	141.5 (119.2, 185.5)	144.5 (86.8, 221.2)	0.83
Hemoglobin (mmol/L)	6.4 (5.6, 7.9)	6.9 (6.2, 8.1)	6.7 (6.0, 7.2)	0.60
Thrombocytes (*10^9^/L)	138.5 (91.0, 202.8)	99.0 (72.0, 137.0)	109.0 (85.5, 132.2)	0.069
Leucocytes (*10^9^/L)	5.3 (4.0, 7.7)	4.2 (3.5, 6.8)	5.2 (3.6, 7.6)	0.59

Data are presented as median (IQR) or as proportions (n) with corresponding percentages (%). Bold *p* values indicate statistical significance. BMI: body mass index; SBP: systolic blood pressure; DBP: diastolic blood pressure; ALD: alcohol-associated liver disease; MASLD: metabolic dysfunction-associated steatotic liver disease; MELD: model for end-stage liver disease; HbA1c: hemoglobin A1c; eGFR: estimated glomerular filtration rate; ALT: alanine aminotransferase; AST: aspartate aminotransferase; GGT: gamma-glutamyl transferase; ALP: alkaline phosphatase.

**Table 3:** Multivariable linear regression analyses demonstrating independent associations of plasma citrate with clinical and laboratory variables in end-stage liver disease patients.

	Model 1		Model 2		Model 3		Model 4	
	Std. β (95% CI)	*p*-Value	Std. β (95% CI)	*p*-Value	Std. β (95% CI)	*p*-Value	Std. β (95% CI)	*p*-Value
Age	0.263 (0.092, 0.434)	0.003	0.111 (−0.087, 0.320)	0.26	0.164 (−0.032, 0.377)	0.097	0.160 (−0.043, 0.381)	0.12
Sex	0.102 (−0.069, 0.273)	0.241	0.012 (−0.175, 0.199)	0.90	0.015 (−0.181, 0.184)	0.99	0.000 (−0.184, 0.184)	1.00
Anti- hypertensives			0.169 (−0.012, 0.358)	0.066	0.141 (−0.038, 0.325)	0.12	0.139 (−0.043, 0.326)	0.13
eGFR			−0.306 (−0.553, −0.130)	0.002	−0.244 (−0.486, −0.059)	0.013	−0.250 (−0.506, −0.052)	0.016
CTP classification								
A					ref		ref	
B					0.327 (0.093, 0.575)	0.007	0.335 (0.077, 0.608)	0.012
C					0.219 (−0.018, 0.457)	0.070	0.233 (−0.065, 0.534)	0.12
MELD score							−0.020 (−0.275, 0.233)	0.87

eGFR: estimated glomerular filtration rate; CTP: Child–Turcotte–Pugh; MELD: the model for end-stage liver disease.

**Table 4:** Cox regression analyses for associations between plasma citrate levels and the risk of all-cause mortality in patients with end-stage liver disease (*n* = 84 men and 45 women).

	Per 1 Ln SD Increment		T1	T2	T3
	HR [95%CI]	*p*-Value		HR [95%CI]	HR [95%CI]
All					
Model 1	1.65 [1.03–2.63]	0.037	Reference	2.00 [0.62–6.41] *p* = 0.219	3.13 [1.03–9.54] *p* = 0.044
Model 2	1.59 [0.97–2.61]	0.065	Reference	1.61 [0.48–5.41] *p* = 0.442	2.79 [0.91–8.54] *p* = 0.072
Model 3	1.49 [0.89–2.48]	0.13	Reference	1.60 [0.47–5.42] *p* = 0.455	2.58 [0.83–7.96] *p* = 0.100
Model 4	1.60 [0.93–2.75]	0.088	Reference	1.95 [0.55–6.93] *p* = 0.303	3.09 [0.94–10.12] *p* = 0.063
Males (*n* = 84, 17 deaths)					
Model 1	2.04 [1.08–3.85]	0.027	Reference	5.23 [0.64–42.64] *p* = 0.122	8.19 [1.02–65.67] *p* = 0.048
Model 2	2.11 [1.06–4.22]	0.034	Reference	4.02 [0.47–34.15] *p* = 0.202	7.44 [0.93–59.82] *p* = 0.059
Model 3	1.94 [0.88–4.26]	0.10	Reference	3.63 [0.41–32.08] *p* = 0.246	6.45 [0.75–55.35] *p* = 0.089
Model 4	1.96 [0.86–4.49]	0.11	Reference	3.51 [0.38–32.09] *p* = 0.266	6.02 [0.67–54.37] *p* = 0.11
Females (*n* = 45, 12 deaths)					
Model 1	1.23 [0.62–2.45]	0.56	Reference	0.85 [0.16–4.53] *p* = 0.851	1.44 [0.34–6.02] *p* = 0.621
Model 2	1.19 [0.58–2.43]	0.63	Reference	0.70 [0.12–4.08] *p* = 0.694	1.31 [0.31–5.56] *p* = 0.71
Model 3	1.17 [0.57–2.40]	0.67	Reference	0.73 [0.12–4.57] *p* = 0.736	1.324 [0.31–5.66] *p* = 0.70
Model 4	1.99 [0.78–5.04]	0.15	Reference	2.62 [0.34–20.18] *p* = 0.35	5.46 [0.66–44.92] *p* = 0.12

HRs are expressed per 1 Ln SD increment. Model 1: crude. Model 2: model 1, plus age and sex (not for sex-stratified analyses). Model 3: model 2, plus adjustment for eGFR. Model 4: model 3, with adjustment for Child–Turcotte–Pugh classification. Bold *p* values indicate statistical significance. ESLD: end-stage liver disease; eGFR: estimated glomerular filtration rate.

## Data Availability

Data are available upon reasonable request.

## Supplementary Materials

The following supporting information can be downloaded at: https://www.mdpi.com/article/10.3390/ijms252312806/s1.

## References

[1] Kaplan A., Fortune B., Ufere N., Brown R.S., Rosenblatt R. (2021). National Trends in Location of Death in Patients with End-Stage Liver Disease. Liver Transpl..

[2] Wahid N.A., Lee J., Kaplan A., Fortune B.E., Safford M.M., Brown R.S., Rosenblatt R. (2021). Medicaid Expansion Association with End-Stage Liver Disease Mortality Depends on Leniency of Medicaid Hepatitis C Virus Coverage. Liver Transpl..

[3] Miro J., Laguno M., Moreno A., Rimola A., Hospital Clinic Olt In Hiv Working Group (2006). Management of end stage liver disease (ESLD): What is the current role of orthotopic liver transplantation (OLT)?. J. Hepatol..

[4] Tapper E.B., Parikh N.D. (2018). Mortality due to cirrhosis and liver cancer in the United States, 1999–2016: Observational study. BMJ.

[5] Mansouri A., Gattolliat C.-H., Asselah T. (2018). Mitochondrial Dysfunction and Signaling in Chronic Liver Diseases. Gastroenterology.

[6] Fromenty B., Roden M. (2023). Mitochondrial alterations in fatty liver diseases. J. Hepatol..

[7] Engelmann C., Clària J., Szabo G., Bosch J., Bernardi M. (2021). Pathophysiology of decompensated cirrhosis: Portal hypertension, circulatory dysfunction, inflammation, metabolism and mitochondrial dysfunction. J. Hepatol..

[8] Amjad W., Shalaurova I., Garcia E., Gruppen E.G., Dullaart R.P.F., DePaoli A.M., Jiang Z.G., Lai M., Connelly M.A. (2023). Circulating Citrate Is Associated with Liver Fibrosis in Nonalcoholic Fatty Liver Disease and Nonalcoholic Steatohepatitis. Int. J. Mol. Sci..

[9] Bourgonje A.R., Connelly M.A., Van Goor H., Van Dijk P.R., Dullaart R.P.F. (2023). Plasma Citrate Levels Are Associated with an Increased Risk of Cardiovascular Mortality in Patients with Type 2 Diabetes (Zodiac-64). J. Clin. Med..

[10] Martínez-Reyes I., Chandel N.S. (2020). Mitochondrial TCA cycle metabolites control physiology and disease. Nat. Commun..

[11] Arnold P.K., Finley L.W.S. (2023). Regulation and function of the mammalian tricarboxylic acid cycle. J. Biol. Chem..

[12] Fischer K., Kettunen J., Würtz P., Haller T., Havulinna A.S., Kangas A.J., Soininen P., Esko T., Tammesoo M.-L., Mägi R. (2014). Biomarker Profiling by Nuclear Magnetic Resonance Spectroscopy for the Prediction of All-Cause Mortality: An Observational Study of 17,345 Persons. PLoS Med..

[13] Otvos J.D., Shalaurova I., May H.T., Muhlestein J.B., Wilkins J.T., McGarrah R.W., Kraus W.E. (2023). Multimarkers of metabolic malnutrition and inflammation and their association with mortality risk in cardiac catheterisation patients: A prospective, longitudinal, observational, cohort study. Lancet Healthy Longev..

[14] Costello L.C., Franklin R.B. (2016). Plasma Citrate Homeostasis: How It Is Regulated; And Its Physiological and Clinical Implications. An Important, But Neglected, Relationship in Medicine. HSOA J. Hum. Endocrinol..

[15] Tong W., Hannou S.A., Wang Y., Astapova I., Sargsyan A., Monn R., Thiriveedi V., Li D., McCann J.R., Rawls J.F. (2022). The intestine is a major contributor to circulating succinate in mice. FASEB J..

[16] Serena C., Ceperuelo-Mallafré V., Keiran N., Queipo-Ortuño M.I., Bernal R., Gomez-Huelgas R., Urpi-Sarda M., Sabater M., Pérez-Brocal V., Andrés-Lacueva C. (2018). Elevated circulating levels of succinate in human obesity are linked to specific gut microbiota. ISME J..

[17] Duong N., Bajaj J.S. (2021). The impact of the gut microbiome on liver transplantation. Curr. Opin. Organ Transplant..

[18] Chen Y., Yang F., Lu H., Wang B., Chen Y., Lei D., Wang Y., Zhu B., Li L. (2011). Characterization of fecal microbial communities in patients with liver cirrhosis. Hepatology.

[19] Stryeck S., Gastrager M., Degoricija V., Trbušić M., Potočnjak I., Radulović B., Pregartner G., Berghold A., Madl T., Frank S. (2019). Serum Concentrations of Citrate, Tyrosine, 2- and 3- Hydroxybutyrate are Associated with Increased 3-Month Mortality in Acute Heart Failure Patients. Sci. Rep..

[20] Sanchez-Gimenez R., Peiró Ó.M., Bonet G., Carrasquer A., Fragkiadakis G.A., Bulló M., Papandreou C., Bardaji A. (2023). TCA cycle metabolites associated with adverse outcomes after acute coronary syndrome: Mediating effect of renal function. Front. Cardiovasc. Med..

[21] Schneider J., Han W.H., Matthew R., Sauvé Y., Lemieux H. (2020). Age and sex as confounding factors in the relationship between cardiac mitochondrial function and type 2 diabetes in the Nile Grass rat. PLoS ONE.

[22] Norheim F., Hasin-Brumshtein Y., Vergnes L., Chella Krishnan K., Pan C., Seldin M.M., Hui S.T., Mehrabian M., Zhou Z., Gupta S. (2019). Gene-by-Sex Interactions in Mitochondrial Functions and Cardio-Metabolic Traits. Cell Metab..

[23] Broussard J.L., Perreault L., Macias E., Newsom S.A., Harrison K., Bui H.H., Milligan P., Roth K.D., Nemkov T., D’Alessandro A. (2021). Sex Differences in Insulin Sensitivity are Related to Muscle Tissue Acylcarnitine But Not Subcellular Lipid Distribution. Obesity.

[24] Von Elm E., Altman D.G., Egger M., Pocock S.J., Gøtzsche P.C., Vandenbroucke J.P. (2014). The Strengthening the Reporting of Observational Studies in Epidemiology (STROBE) Statement: Guidelines for reporting observational studies. Int. J. Surg..

[25] Eisenga M.F., Gomes-Neto A.W., Van Londen M., Ziengs A.L., Douwes R.M., Stam S.P., Osté M.C.J., Knobbe T.J., Hessels N.R., Buunk A.M. (2018). Rationale and design of TransplantLines: A prospective cohort study and biobank of solid organ transplant recipients. BMJ Open.

[26] World Medical Association (2013). World Medical Association Declaration of Helsinki: Ethical Principles for Medical Research Involving Human Subjects. JAMA.

[27] Gruppen E.G., Kunutsor S.K., Kieneker L.M., Van Der Vegt B., Connelly M.A., De Bock G.H., Gansevoort R.T., Bakker S.J.L., Dullaart R.P.F. (2019). GlycA, a novel pro-inflammatory glycoprotein biomarker is associated with mortality: Results from the PREVEND study and meta-analysis. J. Intern. Med..

[28] Kappelle P.J.W.H., Gansevoort R.T., Hillege J.L., Wolffenbuttel B.H.R., Dullaart R.P.F., on behalf of the PREVEND study group (2011). Apolipoprotein B/A-I and total cholesterol/high-density lipoprotein cholesterol ratios both predict cardiovascular events in the general population independently of nonlipid risk factors, albuminuria and C-reactive protein: (Apo)lipoproteins and cardiovascular risk. J. Intern. Med..

[29] Inker L.A., Schmid C.H., Tighiouart H., Eckfeldt J.H., Feldman H.I., Greene T., Kusek J.W., Manzi J., Van Lente F., Zhang Y.L. (2012). Estimating Glomerular Filtration Rate from Serum Creatinine and Cystatin C. N. Engl. J. Med..

[30] Wiesner R., Edwards E., Freeman R., Harper A., Kim R., Kamath P., Kremers W., Lake J., Howard T., Merion R.M. (2003). Model for end-stage liver disease (MELD) and allocation of donor livers. Gastroenterology.

[31] Pugh R.N.H., Murray-Lyon I.M., Dawson J.L., Pietroni M.C., Williams R. (2005). Transection of the oesophagus for bleeding oesophageal varices. Br. J. Surg..

[32] Bedi S., Garcia E., Jeyarajah E., Shalaurova I., Perez-Matos M., Jiang Z., Dullaart R., Matyus S., Kirk W., Otvos J. (2020). Characterization of LP-Z Lipoprotein Particles and Quantification in Subjects with Liver Disease Using a Newly Developed NMR-Based Assay. J. Clin. Med..

