# Supplementary material for: Circulating Citrate Is Reversibly Elevated in Patients with End-Stage Liver Disease: Association with All-Cause Mortality
Source: Int J Mol Sci. 2024 Nov 28;25(23):12806. doi: 10.3390/ijms252312806 (PMC11641540; doi:10.3390/ijms252312806)
Supplement: Supplementary file 1 [file ijms-25-12806-s001.zip › ijms-3295494-supplementary.pdf]

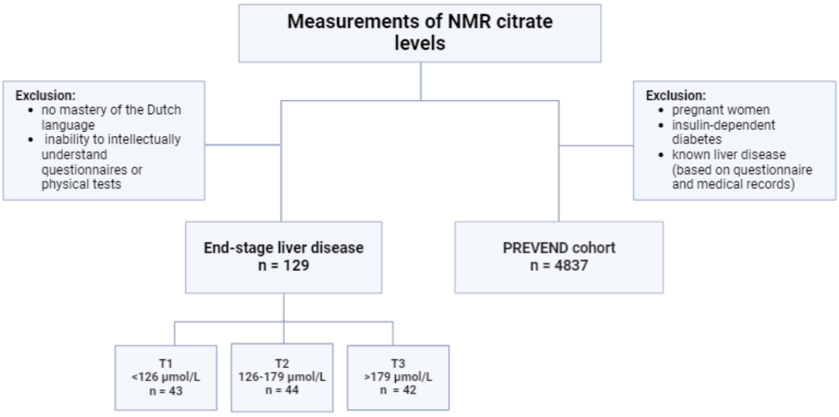


**Supplemental Figure S1** CONSORT flow chart of the study population. NMR: Nuclear magnetic resonance spectroscopy. T1-T3: Tertiles of citrate levels, 1 through 3.
